# Efficacy and safety of artemether-lumefantrine for the treatment of uncomplicated malaria in the setting of three different chemopreventive regimens

**DOI:** 10.1186/s12936-015-0583-9

**Published:** 2015-02-05

**Authors:** James Kapisi, Victor Bigira, Tamara Clark, Stephen Kinara, Florence Mwangwa, Jane Achan, Moses Kamya, Seyi Soremekun, Grant Dorsey

**Affiliations:** Infectious Diseases Research Collaboration, Mulago Hospital Complex, PO Box 7475, Kampala, Uganda; Department of Medicine, San Francisco General Hospital, University of California, San Francisco, CA USA; Department of Pediatrics, Makerere University College of Health Sciences, Kampala, Uganda; Department of Medicine, Makerere University College of Health Sciences, Kampala, Uganda; Faculty of Epidemiology and Public Health, London School of Hygiene and Tropical Medicine, London, UK

**Keywords:** Malaria, Artemether-lumefantrine, Chemoprevention, Sulfadoxine-pyrimethamine, Trimethoprim-sulfamethoxazole, Dihydroartemisinin-piperaquine

## Abstract

**Background:**

The burden of malaria remains high for children in parts of Africa despite the use of insecticide-treated bed nets (ITNs). Chemoprevention has the potential of reducing the malaria burden; however, limited data exist on the efficacy and safety of anti-malarial therapy in the setting of chemoprevention.

**Methods:**

600 children 4–5 months of age were enrolled in Tororo, Uganda, an area of high transmission intensity. Participants were given ITNs, and caregivers instructed to bring their child to a study clinic whenever they were ill. Starting at six months of age, 579 were randomized to no chemoprevention, monthly sulphadoxine-pyrimethamine (SP), daily trimethoprim-sulphamethoxazole (TS), or monthly dihydroartemisinin-piperaquine (DP). Study drugs were administered unsupervised at home until 24 months of age. Episodes of uncomplicated malaria were treated with artemether-lumefantrine (AL) with active follow-up for 28 days. The cumulative risk of recurrent malaria within 84 days and the risk of adverse events within 28 days were compared across study arms using a Cox proportional hazards model and generalized estimating equations, respectively.

**Results:**

A total of 1007, 919, 736, and 451 episodes of malaria were treated in the no chemoprevention, SP, TS, and DP arms, respectively. Only 19 (0.6%) treatments were for severe malaria. Early response to therapy with AL was excellent with 96.5% fever clearance and 99.4% parasite clearance by day 3. However, over 50% of AL treatments were followed by recurrent parasitaemia within 28 days. Compared to the no chemoprevention arm, the cumulative risk of recurrent malaria within 84 days following treatment of uncomplicated malaria with AL was significantly lower in the DP arm (HR = 0.77, 95% CI 0.63-0.95, p = 0.01) but not the SP or TS arms. Compared to the no chemoprevention arm, none of the chemopreventive regimens were associated with an increased risk of adverse events following treatment of malaria with AL.

**Conclusions:**

The risk of severe malaria was very low in this cohort of young children living in a high transmission setting. In the setting of chemoprevention, treatment of uncomplicated malaria with AL was safe and efficacious, with moderate protection against recurrent malaria among children assigned monthly DP.

**Trial registration:**

ClinicalTrials.gov NCT00948896.

## Background

The burden of malaria remains unacceptably high in some parts of sub-Saharan Africa despite the scaling up of control interventions including provision of insecticide-treated bed nets (ITNs), indoor residual spraying of insecticide, and prompt malaria case management with artemisinin-based combination therapy (ACT). In 2012, there were an estimated 207 million cases and 627,000 deaths, with 80% of cases and 90% of deaths occurring in Africa, primarily in children under five years of age [1]. With such high morbidity and mortality, achieving Millennium Development Goals 4 and 6 by 2015 will be unlikely without additional control measures, especially in areas of Africa with persistent high malaria transmission intensity.

The use of anti-malarial drugs for the prevention of malaria in children at high risk has recently received widespread attention as an alternative control strategy. Intermittent preventive therapy in infants (IPTi) with sulphadoxine-pyrimethamine (SP) at the time of routine vaccinations has been recommended by the WHO in certain settings [1]. Alternatively, seasonal malaria chemoprevention (SMC) at monthly intervals generally with combination of SP plus amodiaquine has been recommended by the WHO is parts of West Africa where malaria transmission is primarily limited to a few months during the year [1]. However, alternative drugs and/or approaches are needed in areas where the prevalence of resistance to antifolate drugs is high or malaria transmission occurs throughout the year.

Two parallel randomized control trials were recently completed in cohorts of HIV-unexposed (born to HIV uninfected mothers) and HIV-exposed (born to HIV infected mothers) children aged 6–24 months living in an area of Uganda with high antifolate resistance and intense, year-round transmission [2,3]. Children were randomized to no chemoprevention, monthly SP, daily trimethoprim-sulphamethoxazole (TS), or monthly dihydroartemisinin-piperaquine (DP) for the prevention of malaria. In these trials, monthly DP was highly effective, daily TS moderately effective, and monthly SP provided no significant protection against malaria. In all three treatment arms the burden of uncomplicated malaria remained substantial, requiring frequent treatment with artemether-lumefantrine (AL), the current first-line therapy for uncomplicated malaria in Uganda and many other African countries. The treatment of malaria in the setting of various chemopreventive regimens raises questions regarding the safety and efficacy of AL when other concomitant anti-malarial drugs are being used. In this report, initial response to therapy, the risk of recurrent parasitaemia and recurrent malaria, and the risk of adverse events following the treatment of uncomplicated malaria with AL were compared among children not taking chemoprevention with children taking three different chemoprevention regimens.

## Methods

### Study design, site and population

**Figure 1:**
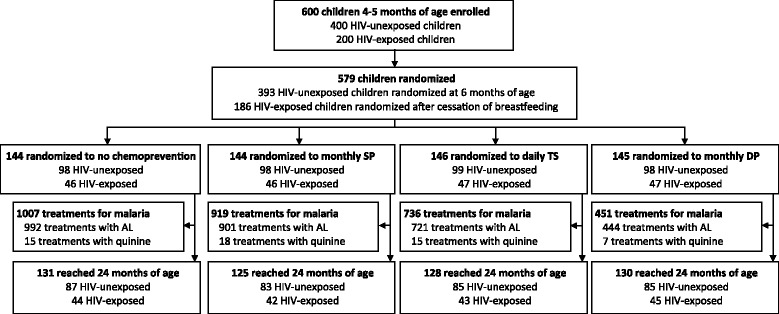
**Study profile.** SP = sulphadoxine-pyrimethamine, TS = trimethoprim-sulphamethoxazole, DP = dihydroartemisinin-piperaquine, AL = artemether-lumefantrine.

Two parallel, open label, randomized control trials of malaria chemoprevention were conducted in Tororo, Eastern Uganda, an area with intense year-round malaria transmission and an entomological inoculation rate (EIR) estimated at 125 infectious bites per person-year in 2011–12 [4]. Details of the parent clinical trials have been reported [2,3]. Briefly, convenience sampling was used to enroll 600 infants “(400 HIV-exposed, 200 HIV-exposed)” 4–5 months of age from the Tororo District Hospital antenatal clinic between June 2010 and July 2011 (Figure 1). At enrollment each household was given two long-lasting ITNs. Study participants were randomized to one of four chemoprevention arms: no chemoprevention, TS (Co-trimoxazole, Kampala Pharmaceutical Industries*,* Uganda) single dose once daily, SP (Kamsidar, Kampala Pharmaceutical Industries*,* Uganda) single dose each month, and DP (Duo-Cotexin, Holley-Cotec, Beijing, China) once daily for three consecutive days each month. HIV-unexposed children were randomized at six months of age and the HIV-exposed were randomized after cessation of breastfeeding and confirmation of their HIV-negative status (median age 10 months). Chemoprevention drugs were administered unsupervised at home according to weight-based guidelines.

### Study procedures

The purpose of this report was to evaluate the safety and efficacy of treatment for malaria in the context of chemoprevention. Participants received all of their medical care at a designated study clinic open every day. Parents/guardians were encouraged to bring their children to the clinic any time they were ill. Children who presented with a documented fever (tympanic temperature ≥38.0°C) or history of fever in the previous 24 hours had blood obtained by finger prick for a thick blood smear. If the smear was positive, the patient was diagnosed with malaria and a complete blood count (CBC) and thin blood smear for parasite speciation were performed. Episodes of uncomplicated malaria were treated with artemether-lumefantrine (AL), the recommended first-line treatment in Uganda. AL was administered twice a day for 3 days, with the first daily dose given with milk and under direct observation in the clinic. The second daily dose was administered at home. Mothers were encouraged to give the second dose with milk or after a meal. Episodes of complicated malaria (severe malaria or danger signs) [5] or treatment failures occurring within 14 days of prior therapy were treated with quinine. All episodes of malaria were actively followed on days 1, 2, 3, 7, 14, 21, and 28 following the diagnosis with repeat thick blood smears on all follow-up days except day 1 and repeat haemoglobin measurement on day 28 or the day of clinical failure. At each follow-up visit study clinicians assessed patients for adverse events according to standardized criteria based on National Institutes of Health guidelines. Serious adverse events were defined as experiences resulting in death, life-threatening experience, inpatient hospitalization, persistent or significant incapacity, or medical or surgical intervention to prevent serious outcomes. Following 28-day active surveillance, children were followed passively until their next diagnosis of malaria or the end of the study. Study participants were followed until they reached 24 months of age or were prematurely withdrawn from the study for any of the following: 1) movement out of the study area, 2) failure to be seen in the study clinic for >60 consecutive days, 3) withdrawal of informed consent, or 4) inability to comply with the study schedule and procedures.

### Laboratory procedures

Thick and thin blood smears were stained with 2% Giemsa for 30 minutes. Parasite density was estimated by counting the number of asexual parasites per 200 white blood cells and assuming a white blood cell count of 8,000 per μL. A thick smear was deemed negative if no parasites were seen in 100 high powered fields. Thin smears were used for species identification. Microscopy quality control included re-reading all blood smears and resolution of any discrepancies by a third microscopist. Haemoglobin levels were measured using a Beckham coulter counter machine or a portable spectrophotometer (HemoCue, Ängelholm, Sweden). Piperaquine (PQ) drug levels were measured from capillary blood collected on filter paper on the day malaria was diagnosed among study participants randomized to monthly DP, as previously described [2].

### Statistical methods

Data were double-entered and verified in Microsoft Access and statistical analyses performed using Stata, version 12 (Stata-Corp). Efficacy and safety outcomes were assessed at the level of each treatment of uncomplicated malaria with AL. 28-day efficacy outcomes included fever and parasite clearance by day 3, appearance of gametocytes not present on day 0, haemoglobin recovery, and WHO treatment outcomes using a standardized classification system [5]. 28-day safety outcomes were based on the identification of adverse events not present on day 0 or of increased severity compared to day 0. These outcomes were compared between the no chemoprevention arm and each of the three chemoprevention regimens using generalized estimating equations with adjustment for repeated measures in the same patient. Cumulative risks of recurrent malaria within 84 days were estimated using the Kaplan-Meier product limit formula with data censored for patients who did not complete follow-up. Univariate and multivariate analyses of risk factors associated with recurrent malaria were made using Cox proportional hazards models with adjustment for repeated measures in the same patient. Risk factors for recurrent malaria of interest included the assigned chemoprevention arm, household wealth index [6], area of residence, age, pre-treatment parasite density, gender, HIV exposure status for all children, and PQ drug levels on the day malaria was diagnosed for children randomized to monthly DP. A p value of <0.05 was considered statistically significant.

### Ethical approval

Ethical approval was obtained from the Uganda National Council for Science and Technology, the Makerere University School of Medicine Research and Ethics Committee, and the University of California, San Francisco Committee on Human Research.

## Results

### Study profile and characteristics of the malaria episodes

A total of 579 (393 HIV-unexposed and 186 HIV-exposed) children were randomized to one of the four chemoprevention arms and 514 (340 HIV-unexposed and 174 HIV-exposed) were followed up to 24 months of age (Figure 1). A total of 3,113 treatments for malaria were given across all 4 arms (97.0% due to *P. falciparum*) of which 3,058 (98.2%) were treated with AL and 55 were treated with quinine (Figure 1). Indications for quinine treatment included 20 with danger signs without meeting criteria for severe malaria, 19 with severe malaria (15 severe anaemia, three multiple convulsions, one respiratory distress), and 16 with recurrent uncomplicated malaria within 14 days of treatment with AL. Baseline characteristics of all episodes of uncomplicated malaria treated with AL stratified by chemoprevention arm are presented in Table 1. Compared to the no chemoprevention arm, children with uncomplicated malaria in the TS and DP chemoprevention arms were significantly older and more likely to be in the lowest tertile of household wealth. Compared to the no chemoprevention arm, children with uncomplicated malaria in the TS chemoprevention arm had significantly lower parasite densities and higher prevalence of gametocytes (Table 1). There were no significant differences across the 4 chemoprevention arms in gender, location of residence, HIV-exposure status, temperature, or haemoglobin levels. Over 95% of parents/guardians reported giving the last assigned dose of chemoprevention drug prior to episodes of uncomplicated malaria being diagnosed.

**Table 1:** **Baseline characteristics of all episodes of uncomplicated falciparum malaria treated with AL**

**Characteristic**	**Chemoprevention arm**			
	**No chemoprevention**	**Monthly**	**Daily**	**Monthly**
		**SP**	**TS**	**DP**
Number of episodes	992	901	721	444
Patient age in months, mean (SD)	16.0 (5.1)	16.1 (5.1)	16.9 (4.8)^b^	17.8 (4.5)^b^
Female gender, n (%)	428 (43.2%)	407 (45.2%)	344 (47.7%)	211 (47.5%)
Urban residence, n (%)	13 (1.3%)	26 (2.9%)	14 (1.9%)	18 (4.1%)
Household wealth index, n (%)				
Lowest tertile	319 (32.2%)	284 (31.5%)	308 (42.7%)^b^	176 (39.6%)^b^
Middle tertile	381 (38.4%)	347 (38.5%)	236 (32.7%)	149 (33.6%)
Highest tertile	292 (29.4%)	270 (30.0%)	177 (24.6%)	119 (26.8%)
HIV-exposed^a^, n (%)	235 (23.7%)	179 (19.9%)	114 (15.8%)	81 (18.2%)
Temperature °C, mean (SD)	38.0 (1.0)	37.9 (1.0)	37.9 (1.0)	37.9 (1.0)
Parasite density per μL, geometric mean	16321	15257	11702^b^	16841
Gametocytes present, n (%)	41 (4.1%)	34 (3.8%)	48 (6.7%)^b^	11 (2.5%)
Haemoglobin g/dL, mean (SD)	9.7 (1.3)	9.6 (1.4)	9.8 (1.3)	9.9 (1.2)
Days since last dose of chemopreventive drug assigned, median (IQR)	N/A	17 (10–24)	1 (1–1)	16 (8–23)
Proportion of prior dose of chemopreventive drug reported taken	N/A	95.5%	98.6%	96.0%

^a^HIV-uninfected children born to HIV-infected mothers.

^b^p-value < 0.05 when compared to no chemoprevention arm.

### Efficacy outcomes

Early response to therapy with AL was excellent across all four chemoprevention arms. There were no significant differences in fever clearance across the chemoprevention arms with over 96% of children afebrile by day 3. There were no significant differences in parasite clearance across the chemoprevention arms with over 92% of blood smears negative by day 2 and over 99% negative by day 3 (Table 2). Only 16 (0.5%) of AL treatments resulted in early treatment failures; 14 developed criteria for severe malaria or dangers signs within 2 days of initiation of AL and 2 had a positive blood smear and fever on day 3. Despite excellent early response to AL therapy, 43.5% of episodes developed recurrent parasitaemia within 28 days of follow-up with no significant differences across the chemoprevention arms. There were no significant differences in the appearance of gametocytes or haemoglobin recovery across the chemoprevention arms (Table 2).

**Table 2:** **Efficacy outcomes after 28 days of follow-up**

**Efficacy outcomes**	**Chemoprevention arm**			
	**No chemoprevention (n = 992)**	**Monthly SP (n = 901)**	**Daily TS (n = 721)**	**Monthly DP (n = 444)**
Fever clearance^a^, n (%)				
Fever present on day 1	497 (50.2%)	445 (49.5%)	339 (47.0%)	203 (46.1%)
Fever present on day 2	73 (7.4%)	87 (9.7%)	58 (8.1%)	32 (7.3%)
Fever present on day 3	37 (3.9%)	32 (3.7%)	20 (2.9%)	16 (3.8%)
Parasite clearance, n (%)				
Positive blood smear on day 2	54 (5.5%)	51 (5.7%)	35 (4.9%)	24 (5.5%)
Positive blood smear on day 3	5 (0.5%)	4 (0.5%)	4 (0.6%)	6 (1.4%)
WHO treatment outcome, n (%)				
No outcome	29 (2.9%)	25 (2.8%)	24 (3.3%)	16 (3.6%)
Early treatment failure	1 (0.1%)	8 (0.9%)	4 (0.6%)	3 (0.7%)
Late clinical failure	172 (17.3%)	148 (16.4%)	112 (15.5%)	71 (16.0%)
Late parasitological failure	284 (28.6%)	240 (26.6%)	190 (26.4%)	115 (25.9%)
Adequate clinical and parasitological response	506 (51.0%)	480 (53.3%)	391 (54.2%)	239 (53.8%)
Appearance of gametocytes^b^, n (%)	33 (3.5%)	33 (3.8%)	24 (3.6%)	16 (3.7%)
Haemoglobin recovery^c^ g/dL, mean (SD)	0.5 (1.3)	0.5 (1.2)	0.6 (1.2)	0.6 (1.1)

^a^Subjective fever over previous 24 hours or temperature ≥ 38.0°C.

^b^Patients with gametocytes present on day 0 not included.

^c^Change in haemoglobin from day 0 to day 28 or day of clinical failure.

**Figure 2:**
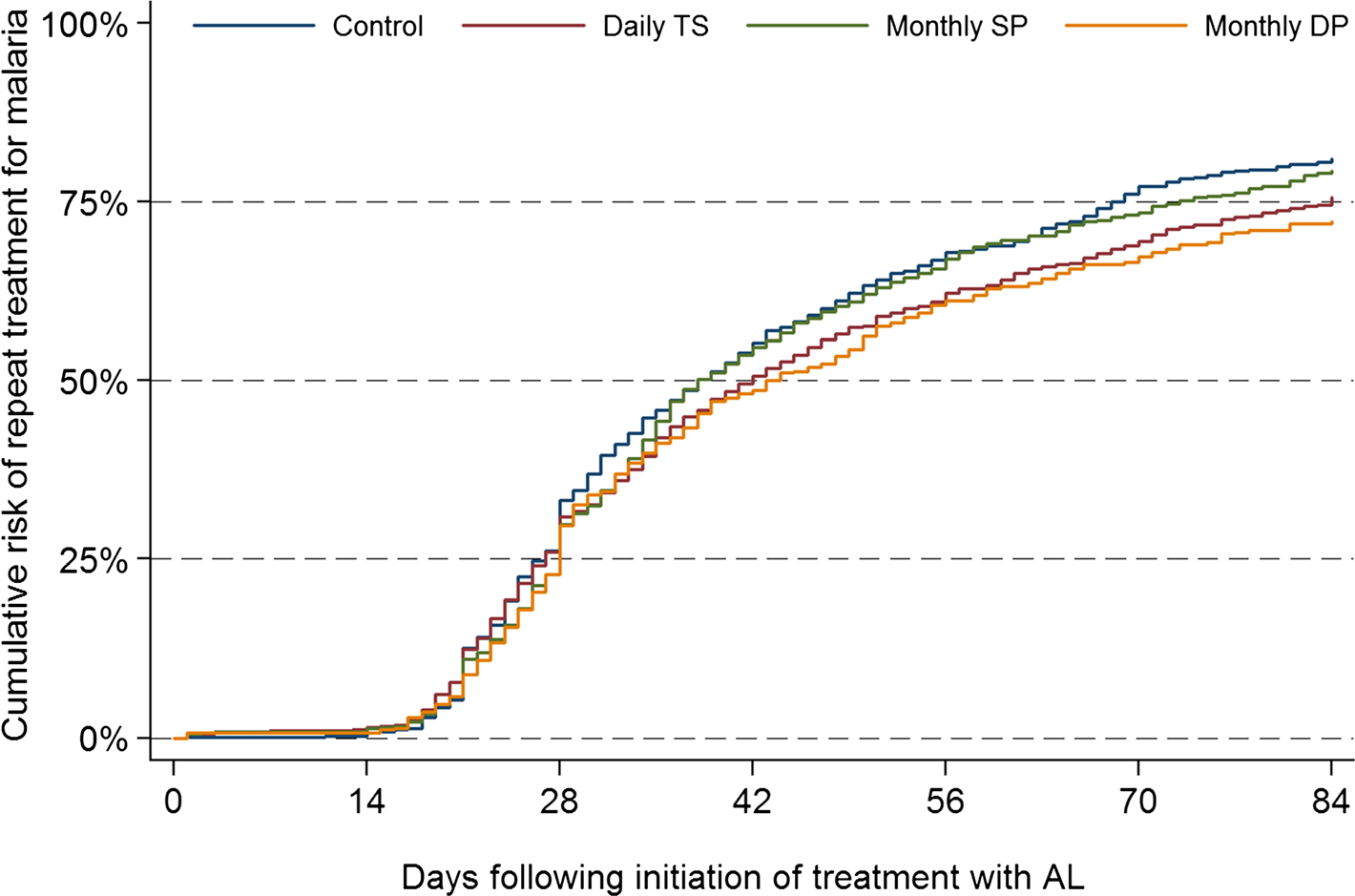
**Cumulative risk of recurrent malaria within 84 days of treatment with artemether-lumefantrine (AL) stratified by chemoprevention arm.**

When follow-up was extended to 84 days following treatment with AL, the cumulative risk of recurrent malaria ranged from 72.2% in the DP chemoprevention arm to 81.0% in the no chemoprevention arm (Figure 2). In multivariate analyses, being assigned to the DP chemoprevention arm was associated with a 23% reduction in the hazard of recurrent malaria compared to the no chemoprevention arm (HR = 0.77, 95% CI 0.63-0.95, p = 0.01). There were no significant differences in the hazard of recurrent malaria between the SP and TS chemoprevention arms compared to the no chemoprevention arm (Table 3). Being in the highest tertile of the wealth index was associated with a significantly lower hazard of recurrent malaria (HR = 0.78, 95% CI 0.66-0.92, p = 0.003), while increasing age (HR = 1.19 per 6 month increase, 95% CI 1.12-1.26, p < 0.001) and higher pre-treatment parasite density (HR = 1.05 per log_10_ increase, 95% CI 1.00-1.10, p = 0.04) were significantly associated with an increased hazard of recurrent malaria (Table 3).

**Table 3:** **Risk factors associated with recurrent malaria within 84 days following treatment with AL**

**Risk factor**		**Univariate analysis**		**Multivariate analysis**	
		**HR** ^**a**^ **(95% CI)**	**p-value**	**HR** ^**a**^ **(95% CI)**	**p-value**
Chemoprevention arm	No chemoprevention	1.0 (reference)	-	1.0 (reference)	-
	Monthly SP	0.95 (0.81-1.11)	0.51	0.95 (0.81-1.12)	0.52
	Daily TS	0.87 (0.72-1.05)	0.14	0.85 (0.70-1.05)	0.07
	Monthly DP	0.81 (0.66-0.99)	0.04	0.77 (0.63-0.95)	0.01
Age (per 6 month increase)		1.15 (1.09-1.23)	<0.001	1.19 (1.12-1.26)	<0.001
Pre-treatment parasite density (per 1og_10_ increase)		1.06 (1.01-1.11)	0.01	1.05 (1.00-1.10)	0.04
HIV-exposure		0.91 (0.77-1.08)	0.27	0.86 (0.73-1.02)	0.09
Female gender		0.94 (0.82-1.07)	0.33	0.94 (0.82-1.07)	0.34
Urban residence		0.57 (0.27-1.20)	0.14	0.65 (0.32-1.33)	0.23
Household wealth index	Lowest tertile	1.0 (reference)	-	1.0 (reference)	-
	Middle tertile	0.92 (0.79-1.07)	0.27	0.91 (0.78-1.06)	0.24
	Highest tertile	0.77 (0.65-0.91)	0.002	0.78 (0.66-0.92)	0.003

^a^Hazard ratio adjusted for repeated measures in the same patient.

**Figure 3:**
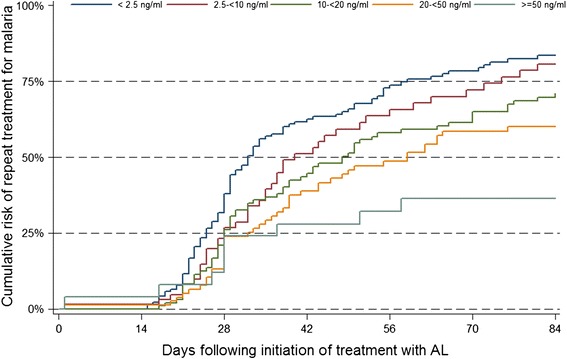
**Cumulative risk of recurrent malaria within 84 days of treatment with artemether-lumefantrine (AL) stratified by pre-treatment piperaquine levels.**

PQ levels were available on the day malaria was diagnosed for 425 of 444 (95.7%) episodes treated with AL among children assigned to chemoprevention with monthly DP. PQ levels were < 10 ng/mL on the day malaria was diagnosed in 51.1% of episodes, suggesting that a complete dose of DP was not administered in the previous month despite the fact that 96.0% of caregivers reported administering the prior dose of DP [7]. There was a strong relationsip between PQ levels at the time malaria was diagnosed and the cumulative risk of recurrent malaria within 84 days following treatment with AL; ranging from 83.5% among episodes with PQ below the level of detection (<2.5 ng/ml) to 36.5% among episodes with PQ levels ≥ 50 ng/ml (Figure 3). Indeed, in multivariate analysis, having a PQ level of ≥ 50 ng/ml was associated with a 70% reduction in the hazard of recurrent malaria (HR = 0.30, 95% CI 0.14-0.63, p = 0.001) compared to those with undetectable PQ levels (Table 4).

**Table 4:** **Associations between PQ levels and risk of recurrent malaria within 84 days following treatment with AL**

**Risk factor**		**Univariate analysis**		**Multivariate analysis** ^**b**^	
		**HR** ^**a**^ **(95% CI)**	**p-value**	**HR** ^**a**^ **(95% CI)**	**p-value**
PQ level (ng/ml) at the time malaria diagnosed	<2.5^c^ (n = 150)	1.0 (reference)	-	1.0 (reference)	-
	2.5 - < 10 (n = 67)	0.80 (0.54-1.17)	0.25	0.83 (0.56-1.24)	0.37
	10 - < 20 (n = 101)	0.65 (0.47-0.89)	0.008	0.67 (0.49-0.93)	0.02
	20 - < 50 (n = 82)	0.50 (0.35-0.72)	<0.001	0.51 (0.36-0.73)	<0.001
	≥50 (n = 25)	0.27 (0.13-0.54)	<0.001	0.30 (0.14-0.63)	0.001

^a^Hazard ratio adjusted for repeated measures in the same patient.

^b^controlling for age, pre-treatment parasite density, HIV-exposure, location of residence, household wealth index.

^c^Below the limits of detection.

### Safety outcomes

Compared to the no chemoprevention arm, none of the chemoprevention arms were associated with an increased risk of common adverse events of any severity following treatment with AL (Table 5). Indeed following treatment with AL, the risks for several common adverse events were significantly lower in those assigned chemoprevention compared to those assigned no chemoprevention: those assigned SP had a significantly lower risk of diarrhoea (21.3 vs 28.8%) and vomiting (9.1% vs 13.5%); those assigned TS had a significantly lower risk of cough (43.6% vs 50.2%), diarrhoea (22.6% vs 28.8%), and anorexia (3.7% vs 6.5%); and those assigned DP had a significantly lower risk of diarrhoea (21.4 vs 28.8%). There were also no significant differences in the risk of any adverse events of severity grade 3–4 or serious adverse events between any of the chemoprevention arms and the no chemoprevention arm. Limiting the analyses to those who were likely to be compliant with their prior dose of DP based on a PQ level of ≥20 ng/ml, there were no significant differences between any of the safety outcomes compared to the no chemoprevention arm (Table 5).

**Table 5:** **Safety outcomes after 28 days of follow-up**

**Safety outcomes**	**Chemoprevention arm**				**Compliant with monthly DP** ^**a**^ **(n = 107)**
	**No chemoprevention (n = 992)**	**Monthly SP (n = 901)**	**Daily TS (n = 721)**	**Monthly DP (n = 444)**	
Common adverse events of any severity, n (%)					
Cough	498 (50.2%)	403 (44.7%)	314 (43.6%)^b^	203 (47.7%)	53 (49.5%)
Elevated temperature	320 (32.3%)	259 (28.8%)	222 (30.8%)	129 (29.1%)	31 (29.0%)
Diarrhoea	286 (28.8%)	192 (21.3%)^b^	163 (22.6%)^b^	95 (21.4%)^b^	26 (24.3%)
Pallor	176 (17.7%)	171 (19.0%)	125 (17.3%)	61 (13.7%)	19 (17.8%)
Vomiting	134 (13.5%)	82 (9.1%)^b^	82 (11.4%)	45 (10.1%)	15 (14.0%)
Anorexia	64 (6.5%)	67 (7.4%)	27 (3.7%)^b^	30 (6.8%)	13 (12.2%)
Any grade 3–4 adverse events, n (%)	87 (8.8%)	95 (10.5%)	52 (7.2%)	38 (8.6%)	13 (12.2%)
Any serious adverse events, n (%)	20 (2.0%)	26 (2.9%)	14 (1.9%)	7 (1.6%)	2 (1.9%)

^a^PQ level ≥ 20 ng/ml at the time treatment with AL initiated.

^b^p-value < 0.05 when compared to no chemoprevention arm.

## Discussion

The use of anti-malarial drugs for the prevention of malaria in children at high risk has been shown to be an effective control intervention and recommended as policy in certain epidemiological settings [1]. In two recently published studies in HIV-unexposed and HIV-exposed Ugandan children 6–24 months of age living in an area of intense, year-round transmission, monthly DP was found to be highly effective, daily TS moderately effective, and monthly SP ineffective for the prevention of malaria [2,3]. However, in the studies the incidence of uncomplicated malaria remained substantial even in the setting of effective chemoprevention requiring frequent treatment with AL, a highly effective ACT widely used in sub-Saharan Africa. This report compares the efficacy and safety of AL in the setting of the 3 chemopreventive regimens described above. Initial response to AL was excellent across all three chemoprevention arms; however recurrent parasitaemia and symptomatic malaria were common within a relatively short period of time following episodes of uncomplicated malaria. Compared to children not assigned chemoprevention, only those assigned chemoprevention with monthly DP had a lower risk of recurrent malaria. In addition, evidence of better compliance with DP based on drug levels measured at the time malaria was associated with better protection against subsequent episodes of malaria. There was no evidence that any of the three chemoprevention regimens was associated with an increased risk of adverse events following treatment of uncomplicated malaria with AL.

Data from this study comes from two parallel randomized control trials in young HIV-unexposed and HIV-exposed children living in a holoendemic area where the incidence of malaria was remarkably high despite the provision of long-lasting ITNs to all study participants. Although over three thousand treatments were given for malaria, over 98% were uncomplicated cases treated with AL. The very low rate of treatment for complicated malaria seen in this study can likely be attributed to the high quality of care and provision of prompt and effective therapy in a research setting. AL is the recommended first-line treatment for uncomplicated malaria in a majority of countries in sub-Saharan Africa including Uganda. AL has been shown to be highly effective with PCR-corrected cure rates above 95% in the majority of studies [8]. AL has also been shown to be safe and well-tolerated with a safety profile comparable to other commonly used ACT [9]. Despite the widespread use and excellent efficacy and safety profile of AL, there is limited data on its use in the setting of chemoprevention. In a cluster randomized trial of seasonal malaria chemoprevention using SP plus amodiaquine in Senegal, treatment of uncomplicated malaria with AL was found to be safe and well tolerated, although results were not stratified as to whether children treated with AL were exposed to chemopreventive drugs [10]. As the use of anti-malarial drugs for chemoprevention is being increasingly advocated for in certain epidemiological settings and being evaluated in others, more data is needed to evaluate the efficacy and safety of anti-malarial drugs used for treatment when other drugs are being used concomitantly for prevention.

In this study, initial response to AL therapy for uncomplicated malaria was excellent, with rapid clearance of fever and parasitaemia, consistent with other studies from Uganda [11,12]. Despite rapid clearance of parasitaemia, the risk of recurrent parasitaemia within 28 days was relatively high and a majority of patients developed recurrent malaria within 84 days. Although genotyping to distinguish recrudescence from new infections was not performed in this study, in another study done a few years earlier at the same study site only 1 of 200 episodes of recurrent malaria following treatment with AL was due to recrudescence (true treatment failure) [13]. Thus it was assumed that the vast majority of episodes of recurrent malaria following treatment with AL in this study were due to new infections. Compared to children not assigned chemoprevention, the risk of recurrent malaria following treatment with AL was similar in children assigned chemoprevention with monthly SP, consistent with the lack of protective efficacy for this treatment arm in the parent clinical trials [2,3]. In the parent clinical trials, chemoprevention with daily TS provided modest protection against malaria, however, in this study there was only a non-significant trend towards a lower risk of recurrent malaria following treatment with AL compared to children assigned to no chemoprevention. In this study, there was no objective measure of adherence to SP or TS, therefore one cannot rule out non-compliance as playing a significant role in these findings. Indeed, it is likely that children who were non-compliant with their chemopreventive drugs would have been at highest risk of developing malaria. In contrast, children who were assigned monthly DP had a significantly lower risk of developing recurrent malaria following treatment with AL and this benefit was greatest among children who had objective evidence of compliance with their study drug based on PQ drug levels measured at the time malaria was diagnosed. These findings suggest that chemoprevention with an effective drug like DP may have the added benefit of reducing the risk of recurrent malaria shortly after treatment for breakthrough episodes of malaria. Indeed, evidence exists that anti-malarial drugs with different modes of action can profoundly influence parasite genetics and drug sensitivity. In a separate study from the same site, recent treatment with AL selected for parasites with *pfmdr1* alleles associated with decreased sensitivity to lumefantrine in subsequent episodes, while recent treatment with DP selected for parasites with the opposite alleles associated with increased sensitivity to lumefantrine [13]. This raises the possibility that the use one ACT for chemoprevention and another ACT for treatment, where the partner drugs have different modes of action, may enhance each other’s efficacy while reducing the risk of propagation of drug resistant parasites.

Perhaps of equal or greater importance than the efficacy of AL, is its safety and tolerability in the setting of concomitant use of other anti-malarial drugs for chemoprevention. In a previous study from Uganda AL was found to be safe and well tolerated with no increased risk of common adverse events among HIV-infected and HIV-exposed children who were prescribed concomitant therapy with daily TS prophylaxis [14]. In this study none of the chemoprevention arms were associated with an increased risk of adverse events within 28 days of treatment with AL compared to the no-chemoprevention arm. Of particular concern was the administration of two ACT regimens both containing artemisinin derivatives (monthly DP for chemoprevention and AL for breakthrough episodes of malaria). When data from this study was limited to children with PQ levels ≥ 20 ng/ml at the time treatment with AL was initiated (consistent with DP being administered in the prior month), the risk of adverse events was no different from children who were not assigned chemoprevention. These findings are reassuring and consistent with a recent large clinical trial conducted in 10 countries where patients with uncomplicated falciparum malaria were treated with three days of oral artesunate (either 2 mg/kg or 4 mg/kg per day) followed by a standard three-day course of an ACT (primarily AL or DP) [15]. This prolonged course was found to be safe and highly effective and may provide an alternative option for treating artemisinin-resistant malaria.

There were several limitations to this study. Foremost was the lack of directly observed therapy, as chemopreventive drugs were administered at home and only the 1st of each daily dose of AL was administered in the study clinic. Additionally, the lack of pharmacokinetic data, with the exception of PQ levels measured at the time malaria was diagnosed in the DP chemoprevention arm, precluded the ability to assess drug-drug interactions and their potential impact on the efficacy and safety of AL. Study investigators, participants, and their parents/guardians were not blinded to assigned chemoprevention regimens, which could have introduced bias, especially with respect to measures of drug safety and tolerability which were largely subjective by nature. Finally, comparisons were made between the three chemoprevention arms and the no chemoprevention arm for multiple efficacy and safety outcomes, resulting in multiple tests of significance, increasing the probability of finding significant differences just by chance.

## Conclusions

In this study conducted in young children living in a high transmission area, over 3,000 episodes of uncomplicated malaria were treated with AL in the setting of three different chemopreventive regimens. AL was found to be safe and effective, with modest protection against recurrent episodes of malaria among children assigned chemoprevention with monthly DP. The use of drugs for the prevention of malaria offers great promise for high risk populations living in Africa. However, breakthrough episodes of malaria will inevitably occur and a better understanding of the optimal management of such episodes in the setting of chemoprevention is needed.

## Abbreviations

AL: Artemether-lumefantrine
TS: Trimethoprim-sulfamethoxazole
SP: Sulfadoxine-pyrimethamine
DP: Dihydroartemisinin-piperaquine
PQ: Piperaquine
IPT: Intermittent preventive therapy
SMC: Seasonal malaria chemoprevention
